# C3-Luc Cells Are an Excellent Model for Evaluation of Cellular Immunity following HPV16L1 Vaccination

**DOI:** 10.1371/journal.pone.0149748

**Published:** 2016-02-22

**Authors:** Li-Li Li, He-Rong Wang, Zhi-Yi Zhou, Jing Luo, Xiao-Li Wang, Xiang-Qian Xiao, Yu-Bai Zhou, Yi Zeng

**Affiliations:** 1 Beijing Key Laboratory of Environmental and Viral Oncology, College of Life Science and Bio-Engineering, Beijing University of Technology, Beijing, China; 2 National Institute for Viral Disease Control and Prevention, Chinese Center for Disease Control and Prevention, State Key Laboratory for Infectious Disease Prevention and Control, Beijing, China; Brigham and Women's Hospital/Harvard Medical School, UNITED STATES

## Abstract

C3 and TC-1 are the two model cell lines most commonly used in studies of vaccines and drugs against human papillomavirus (HPV) infection. Because C3 cells contain both the HPV16 E and L genes, but TC-1 cells contain only the HPV16 E genes, C3 cells are usually used as the model cell line in studies targeting the HPV16 L protein. However, expression of the L1 protein is difficult to detect in C3 cells using common methods. In our study, Short tandem repeat analysis (STR) was used to demonstrate that C3 cells are indeed derived from mice, PCR results show that HPV16 L1, E6 and E7 genes were detected in C3 genomic DNA, and RT-PCR results demonstrated that L1 transcription had occurred in C3 cells. However, the expression of C3 protein was not found in the results of western blot and immunohistochemistry (IHC). Growth and proliferation of C3 were inhibited by mice spleen lymphocytes that had been immunized with a vaccine against HPV16L1. The luciferase gene was integrated into C3 cells, and it was confirmed that addition of the exogenous gene had no effect on C3 cells by comparing cell growth and tumor formation with untransformed cells. Cells stably expressing luciferase (C3-luc) were screened and subcutaneously injected into the mice. Tumors became established and were observed using a Spectrum Pre-clinical in Vivo Imaging System. Tumor size of mice in the different groups at various time points was calculated by counting photons. The sensitivity of the animals to the vaccine was quantified by statistical comparison. Ten or 30 days following injection of the C3-luc cells, tumor size differed significantly between the PBS and vaccine groups, indicating that C3 cells were susceptible to vaccination even after tumors were formed *in vivo*.

## Introduction

Therapeutic vaccines against high-risk human papillomavirus (HPV) are essential for the removal of infected cells and associated cancer cells. Most studies of HPV-induced tumors focus on the E6 and E7 genes, because they are oncogenes and are thus most relevant to carcinoma [1, 2]. Studies in which E6/E7 was the main antigen have focused on abolishing carcinogenic activity while retaining the immunogenicity of the vaccine [3, 4]. To evaluate the effect of using E6/E7 as the core antigen, the model cell line TC-1 was constructed by a research group at Johns Hopkins University in 1996 [5]. This cell line was derived from primary epithelial cells from C57BL/6 mice co-transformed with the E6 and E7 genes of HPV16 and c-Ha-ras oncogenes. The development of this cell line greatly promoted the study of vaccines for treatment of HPV infection and related tumors. Infection with high-risk strains of HPV is known to be involved in several human cancers in addition to cervical cancer, including anogenital tumors, oral cancer, and head and neck squamous cell carcinomas [6–10]. Prophylactic vaccines primarily use the HPV late (L) genes, which encode the major viral capsid proteins L1 and L2 for assembly of virus-like particles (VLP), as the main antigen [11]. Accumulating evidence shows that the L1 early protein is expressed in HPV infection lesions and tumor cells [12, 13]. L1 has become an important antigen for therapeutic vaccines because it can also stimulate an effective cellular immune response [14]. The C3 tumorigenic cell line was constructed by a research group at the Netherlands Leiden Medical School in 1993. The C3 cell line was constructed by transfection with a plasmid containing the full-length HPV 16 genome, including the early (E) and late (L) genes, under control of their homologous enhancer-promoter and co-transfection with activated *ras* into B6 MEC C57BL16 mouse embryo cells[15]. Some previous studies have used C3 cells to evaluate vaccines based on HPV16L1 [14, 16]; however, the L genes, especially L1 gene expression and the immune characteristics of the model cells, have not been adequately studied. Therefore, in this study, we determined whether the HPV16L1 gene is present and its protein is expressed in C3 cells. We recorded the progression of C3 cytolysis by mouse lymphocytes that had been immunized with a vaccine based on HPV16L1. In addition, to facilitate the evaluation of the immune effect of the vaccine, we constructed C3-luc reporter cells by integrating the luciferase gene into C3 cells.

## Materials and Methods

### Cell culture

C3 cells were kindly provided by Zeng Yi, National Institute for Viral Disease Control and Prevention, Chinese Center for Disease Control and Prevention. C3 and C3-luc were maintained in Dulbecco’s modified Eagle medium (DMEM; Gibco, Carlsbad, CA) supplemented with 5% fetal bovine serum (FBS), 100 U/ml of penicillin, 100 mg/ml of streptomycin, and incubated at 37°C in 5% CO_2_.

### Mice

Six-to-eight-week-old female specific-pathogen-free (SPF) C57BL/6 mice were purchased from Beijing Weitong Lihua Experimental Animal Technology Co. Ltd., (Beijing, China) and maintained under pathogen-free conditions at the animal facilities of the Peking University First Hospital. Mice were sacrificed by dislocated spine method under anesthesia (ether). All animal experimental procedures in this study were approved by the animal ethics committee of Peking University First Hospital.

### PCR

PCR was performed to confirm the presence of the HPV16L1 gene in C3 cells. Cells were grown in 25-cm^2^ culture flasks to 80% confluence, and then trypsinized and collected by centrifuging for 5 min at 500 × *g* at 25°C. Genomic DNA was extracted using a MiniBEST Universal Genomic DNA Extraction Kit (TaKaRa 9765). Primers for amplification were designed and synthesized based on the full-length sequences of HPV16 L1, E6, E7 and β-actin, and their sequences are as follows: Forward L1: 5′-ATGTCTCTTTGGCTGCC-3′; Reverse L1: 5′-GTAGAGGTAGATGAGGTGGTGG-3′; Forward E6: 5′-ATGTTTCAGGACCCACAG-3′. Reverse E6: 5′-TTACAGCTGGGTTTCTCTAC-3′. Forward E7: 5′-ATGCATGGAGATACACCTAC-3′. Reverse E6: 5′-TTATGGTTTCTGAGAACAGAG-3′. PCR reactions were performed with genomic DNA of C3 and TC-1 cells as templates. Genomic DNA of TC-1 cells, which have genes for HPV16 E6 and E7, was used as a positive control for the PCR reactions. β-actin Reverse: 5′-AACAGTCCGCCTAGAAGCAC-3′. PCR reactions were performed using genomic DNA of C3 and TC-1 cells as the template. Genomic DNA from TC-1 cells was used as the template for control reactions and β-actin was used as an internal reference. PCR products were separated on a 1.2% agarose gel.

### Reverse-transcription PCR (RT-PCR)

RT-PCR was used to amplify HPV16L1 mRNA. Total RNA was extracted using a MiniBEST Universal RNA Extraction Kit (TaKaRa 9767) and reverse-transcribed using the Quant One Step RT-PCR kit (TIANGEN China KR113). Two pairs of primers were designed: forward primer rtpcr-3′-f: 5′-TTTAATAGGGCTGGTA- CTGTTGG, reverse primer rtpcr-3′-r: 5′-TAGGTGCTGGAGGTGTATGTTTT. Forward primer fullrtpcr-f:5′-ATGAGCCTGTGGCTGCCCAGCG

Reverse primer fullrtpcr-r: 5′-TCACAGCTTCCTCTTCTTCCTCTTGGCGG

Total RNA from C3 cells was used as a template and total RNA from TC-1-HPV16L1 was used as a positive control. PCR reactions using pCDNA3.1-HPV16L1 as the template represented another positive control. The amplified products were subjected to electrophoresis on a 1.2% agarose gel.

### Short tandem repeat (STR) analysis

Genomic DNA was extracted using a MiniBEST Universal Genomic DNA Extraction Kit (TaKaRa 9765). The mouse genotyping Kit (mouse STR V2.0) was used for multiplex PCR reactions of 12 loci, including 18–3, 9–2, 6–7, 5–5, X-1, 15–3, 12–1, 6–4, csf1po, VWA, 4–2, Jarid1, etc. STR loci were detected on a 3730xl ABI genetic analyzer.

### Western Blot

C3 cells were collected by trypsinization and centrifugation. After washing cells with phosphate buffered saline (PBS), cells were mixed with sample buffer and kept in boiling water for 5 min, and then centrifuged at 12,000 × *g* for 5 min. Protein lysate samples were separated on a 10% (v/v) SDS-PAGE gel and transferred onto a nitrocellulose membrane (Bio-Rad, Hercules, CA, USA). Membranes were blocked with 5% (w/v) nonfat dry milk in TBST (10 mM Tris-HCl, pH 7.5, 150 mM NaCl, and 0.05% Tween® 20) overnight at 4°C to reduce non-specific binding. Membranes were incubated with primary anti-HPV16L1 antibody (Abcam, Cambridge, UK; ab 69) followed by an incubation with anti-rabbit IgG-horseradish peroxidase antibody (Beyotime, Haimen, China; A0208). Bound antibodies were detected using a DAB Horseradish Peroxidase Color Development Kit (Beyotime; P0202).

### Cloning and transfection of the pcDNA3.1-luc plasmids

A full-length luciferase open reading frame (ORF) was inserted in-frame into the pcDNA 3.1/blasticidin expression plasmid (constructed and maintained by our laboratory) by PCR amplification using the *Eco*RI and *Not*I restriction sites. Primer sequences were as follows: forward primer P-luc-1: 5′-CCGGAATTCATGGAAGATGCCAAAAACATTAAG, reverse primer P-luc-2: 5′-GCGGCCGCTTACACGGCGATCTTGCCGCCCTTC. The luciferase gene in pGL4.20-luc was used as the template. The pcDNA3.1/blasticidin-luc vector was then transfected into C3 cells using the FuGENE HD Transfection Reagent (Roche 04709691001). Cells were passaged 24 h after transfection, and when the cells covered 50% of the six-well plates the medium was replaced with DMEM containing blasticidin (500 μg/mL). Cells were incubated at 37°C in a 5% CO_2_ atmosphere and the medium was changed once every three days. On days 8, 16, and 24, 1 × 10^4^ cells were counted, serially diluted two-fold (ranging from 2 ^−1^ to 2 ^−5^), and then the cells were transferred to black 96 wells plates and cells were permitted to adhere overnight. D-luciferin was added to the medium at 1/10 volume at a final concentration of 150 μg/mL, and 2 min after addition the results were observed on the IVIS Spectrum Pre-clinical In Vivo Imaging System (IVIS, Clod Spring Biotech Corp).

### Confirmation of the stability of the exogenous gene

C3-luc cells were cultured in DMEM without blasticidin and passaged, and 5^th^ and 10^th^ generation cells were counted after trypsinization and resuspended in DMEM. Several gradients of 2-fold serial dilution were performed with 10^5^ cells/mL. Cells were added to black 96-well plates at 100 μL/well with D-luciferin, at a final concentration of 150 μg/mL, and incubated at room temperature for 2 min before the results were observed on the IVIS. The same method was used to determine luciferase activity in order to evaluate the stability of the luciferase expression level.

### Effect of luciferase on the growth of C3 cells

C3 or C3-luc cells (5 × 10^3^ cells per well) were added to the wells of E-Plate L8 disposable plates (ACEA Biosciences 00300600840) that can be used with the RTCA iCELLigence (ACEA Biosciences00380600970) for dynamic testing of cell function. Plates were inserted into the iCELLigence system and the adherence rate (cell index, CI) of the cells was monitored for 72 h, then the results were exported for data analysis.

### *In vitro* killing test

**Fig 1:**
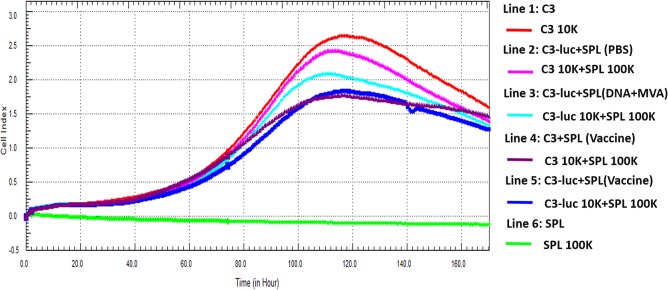
Results of *in vitro* killing experiments indicated by cell growth curves. 1×10^4^ cells of C3 and 1×10^6^ mouse spleen lymphocytes were added to two wells of E-platel8 as controls. Mixtures of C3-luc and different sources of spleen lymphocytes (vaccine group, PBS group and DNA/MVA without HPV16 L1 group) were added to another three wells, and to the last well, a mixture of C3 and SPL of the vaccine group were added.

The spleens were removed from mice after killing the animals under anesthesia (ether) that had been primed with pVR16-HPV16L1 (DNA vaccine) and boosted with MVA-HPV16L1 (virus vaccine) and which had been shown to produce a valid cellular immune response by Elispot testing. Spleen tissues were ground in 5 mL PBS and passed through a 300-mesh screen, then centrifuged at 400 × *g* for 10 min. The supernatant was discarded and the pellet was resuspended in 5 mL of red blood cell lysis buffer (RT122, Tiangen, Beijing, China). Cells were incubated for 5 min and re-pelleted by centrifuging for 10 min at 400 × *g*. Cells were resuspended in 5 mL of DMEM and counted. C3 or C3-luc were added as targets cells and splenic lymphocytes were added as killing cells into the wells of the E-Plate L8, along with a peptide (Sequence: AGVDNRECI, 10 μg/well) that had been proven to induce cellular immunity efficiently (splenic lymphocytes and the peptide were added following overnight culture of C3). For numbers of cells and other specific details, see Fig 1. The plates were inserted into the iCELLigence system and the adherence rate of the cells was monitored for 168h; the data were then exported for analysis.

### Construction of the mouse tumor model

**Fig 2:**
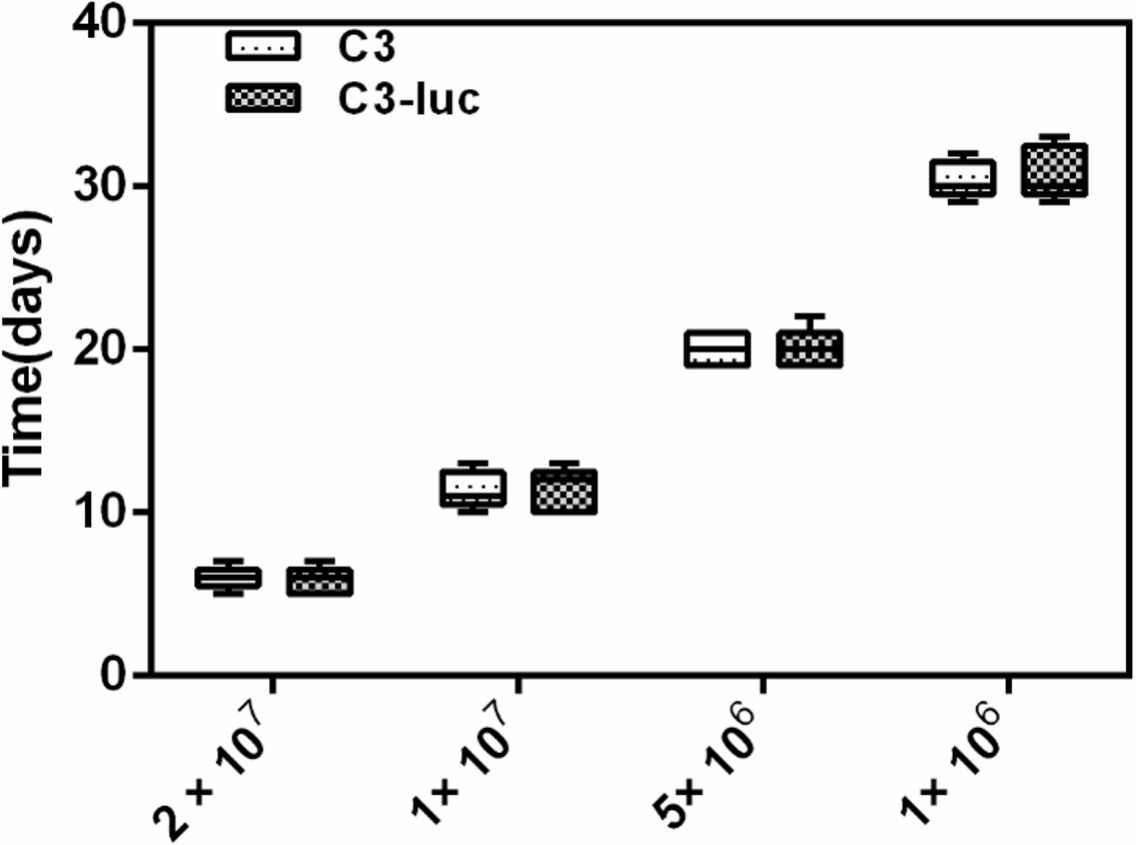
Time required for tumor formation by different numbers of C3 or C3-luc cells *in vivo*. Fig 2 shows that the greater the number of cells inoculated, the faster the tumors were generated. However, time to tumor formation showed no significant differences between mice inoculated with the same number of C3 or C3-luc cells.

Fifty female C57BL/6 mice (6 to 8 weeks old) were selected and divided into 10 groups. C3 or C3-luc cell were subcutaneously injected into the groins of the mice. Animal grouping and the number of cells inoculated are shown in Fig 2. Mice were monitored for the appearance of tumors and the time of appearance was recorded. Three mice inoculated with 2 × 10^7^ C3-luc were imaged after 20 or 30 days using the IVIS.

### Immunohistochemistry

Indicated numbers of tumor cells (1×10^7^ C3, 1×10^7^ C3-luc, 1×10^6^ TC-1, 1×10^6^ TC-1-HPV16L1) were injected into the groin of C57BL/6 mice subcutaneously. After 20 d, tumors were isolated and fixed with multiple formaldehyde treatments. HPV16 L1 expression was immunohistochemically detected in tumor tissues. Expriments of Immunohistochemistry were completed by Beijing Ding Guo Changsheng Biological Technology Co., Ltd.

### Tumor challenge experiments

Ten female C57BL/6 mice were selected and divided into two groups. One group was primed with the pVR16-HPV16L1 (DNA vaccine) and boosted with the MVA-HPV16L1 (virus vaccine); the other group was injected with the same volumes of PBS. One week after vaccination, 2 × 10^7^ C3-luc cells were subcutaneously inoculated into the groins of the mice. Tumors were observed at 10 and 30 days in the IVIS imaging chamber.

### Mouse imaging

Luciferase signal was detected by imaging of whole animals using the *in vivo* IVIS imaging system (Caliper Life Sciences, Runcorn, UK). For firefly luciferase imaging, an intraperitoneal injection of chloral hydrate (10%, 100 μL/mouse) was used to anesthetize the mice. D-luciferin (AAT Bioquest, AAT-12506) at a concentration of 150 mg/kg in sterile PBS was injected after they were fully anesthetized. Animals were imaged for 180 seconds at a binning value of 8 and an FVO of 12.8 cm, 10 minutes after the injection of D-luciferin. Quantification of the bioluminescence signal was performed using the Living Image 4.2 software (CaliperLife Sciences). The measurements are expressed as the total flux of photons per second of imaging time.

### Statistical analysis

All statistical analysis was performed with the GraphPad Prism 6.0 software (GraphPad Software Inc.). Statistical analysis of photon levels was performed using an unpaired *t*-test with Welch’s correction for unequal variance.

## Results

### HPV16 virus genes in C3 genome

**Fig 3:**
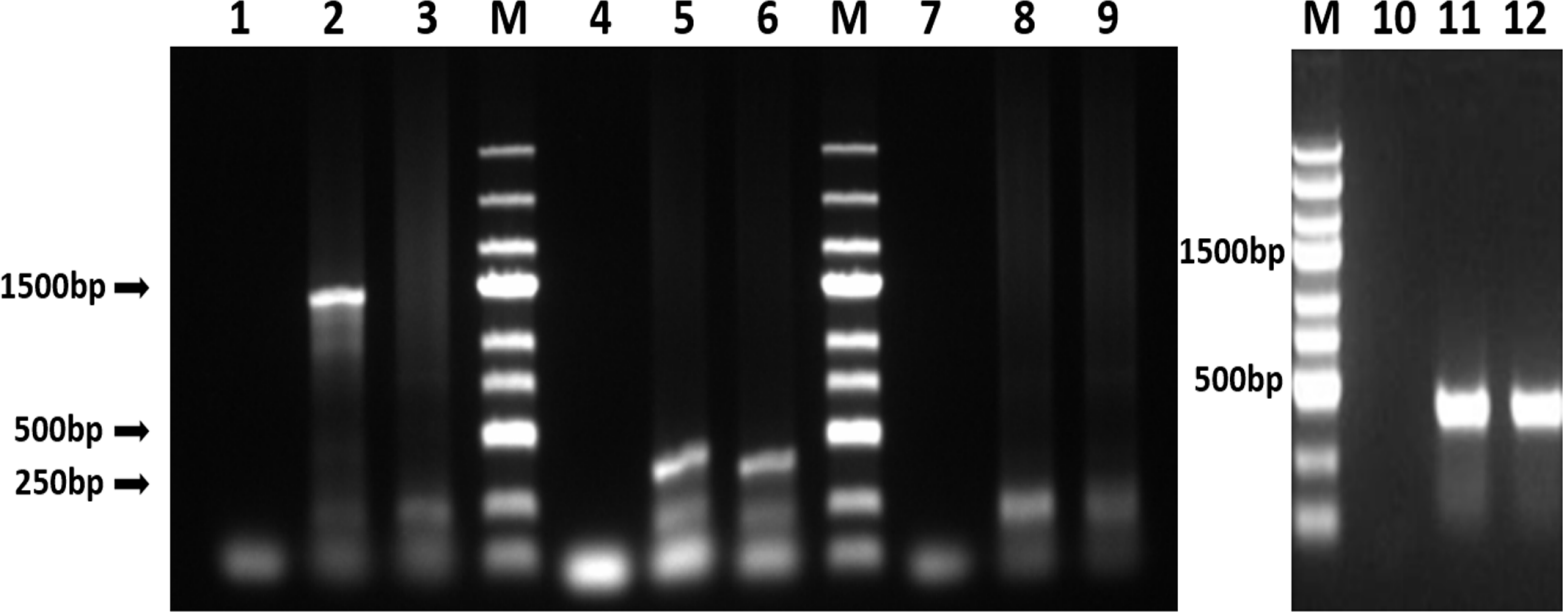
PCR results of HPV16 gene amplification using C3 genomic DNA. Genomic DNA of TC-1 cells were used as a template of control and β-actin was used as an internal reference. M: DNA ladder; Line 1, 4, 7, 10: negative control, Line 2, 5, 8, 10: genomic DNA of C3 cells as template; Line 3, 6, 9, 12: genomic DNA of TC-1 cells as template. Line 1, 2, 3: PCR using primers for HPV16 L1; Line 4, 5, 6: PCR using primers for HPV16 E6; Line 7, 8, 9: PCR using primers for HPV16 E7; Line 10, 11, 12: PCR using primers for β-actin. 1487-bp, 456-bp, 297-bp, and 416-bp fragments were amplified for HPV16 L1, HPV16 E6, HPV16 E7, and β-actin.

HPV16 L1, E6 and E7 genes were detected in C3 genomic DNA. A 1487-bp sequence of the HPV16 L1 gene, a 456-bp sequence of the HPV16 E6 gene and a 293-bp sequence of the HPV16 E7 gene were amplified using C3 genomic DNA as template. (Fig 3). TC-1 genomic DNA was used as a control for the amplification of HPV16 E6 and E7 sequences.

### Expression of HPV16 L1 protein in C3

**Fig 4:**
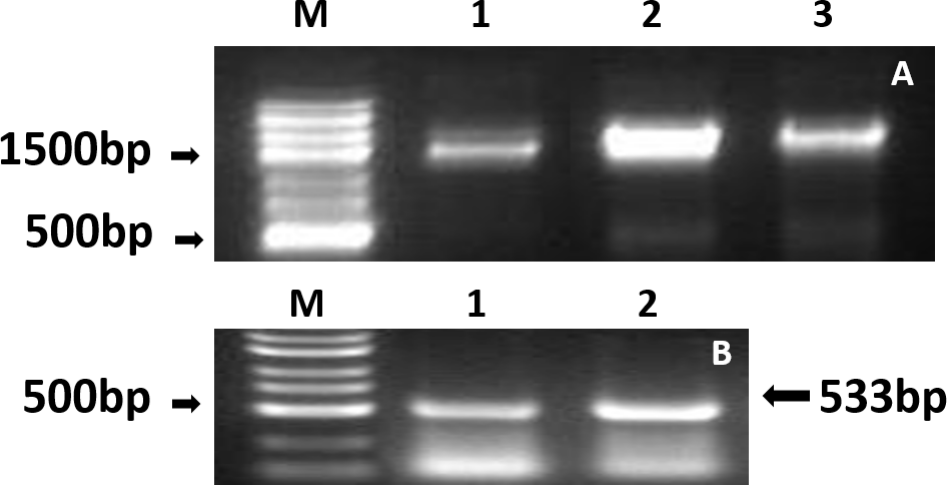
Results of RT-PCR to verify L1 protein synthesis. Total RNA was extracted using a MiniBEST Universal RNA Extraction Kit (TaKaRa 9767) and reverse-transcribed using the Quant One Step RT-PCR kit (TIANGEN China KR113). Two pairs of primers were designed. (A) Results of rt-PCR to amplify the whole sequence of HPV16L1. (B) Results of amplification of the 3′ end of HPV16L1. Fig 4-A: M: DNA ladder; 1: RT-PCR result using genomic DNA of C3 as template; 2: PCR result using plasmid pCDNA3.1-HPV16 L1 as template; 3: RT-PCR result using genomic DNA of TC-1-HPV16 L1 as template; Fig 4-B: M: DNA ladder; 1: RT-PCR result using genomic DNA of C3 as template; 2: RT-PCR result using genomic DNA of TC-1-HPV16 L1 as template.

For amplification of mRNA, two pairs of primers were designed, with which the full-length HPV16L1 sequences (Fig 4A) and a 533-bp sequence (Fig 4B) of the 3′ end of the L1 gene were amplified, from bp 859 to bp 1387 of HPV16L1. Together these results indicate positive transcription of the HPV16 L1 gene in C3 cells.

**Fig 5:**
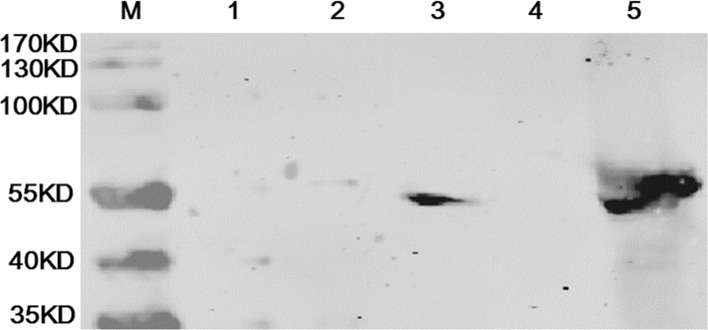
HPV16 L1 protein was not detected in C3 by western blot analysis M: protein marker; 1: C3; 2: TC-1; 3: TC-1-HPV16L1; 4: BHK-21; 5: BHK-21 infected with MVA-HPV16 L1. Lines 3 and 5 represent positive controls and line 2 and 4 represent negative controls.

Protein samples from five cells (C3, TC-1, TC-1-HPV16L1, BHK-21, BHK-21 infected by MVA-HPV16L1) were prepared for western blot analysis. TC-1-HPV16 L1 and BHK-21 infected with MVA-HPV16 L1 were used as positive controls for the expression of HPV16 L1. TC-1 and BHK-21 cells, which do not express HPV16 L1, were used as negative controls. Western blot analysis results demonstrate that HPVL1 protein was not expressed in C3 cells. Western blot results are shown in Fig 5.

**Fig 6:**
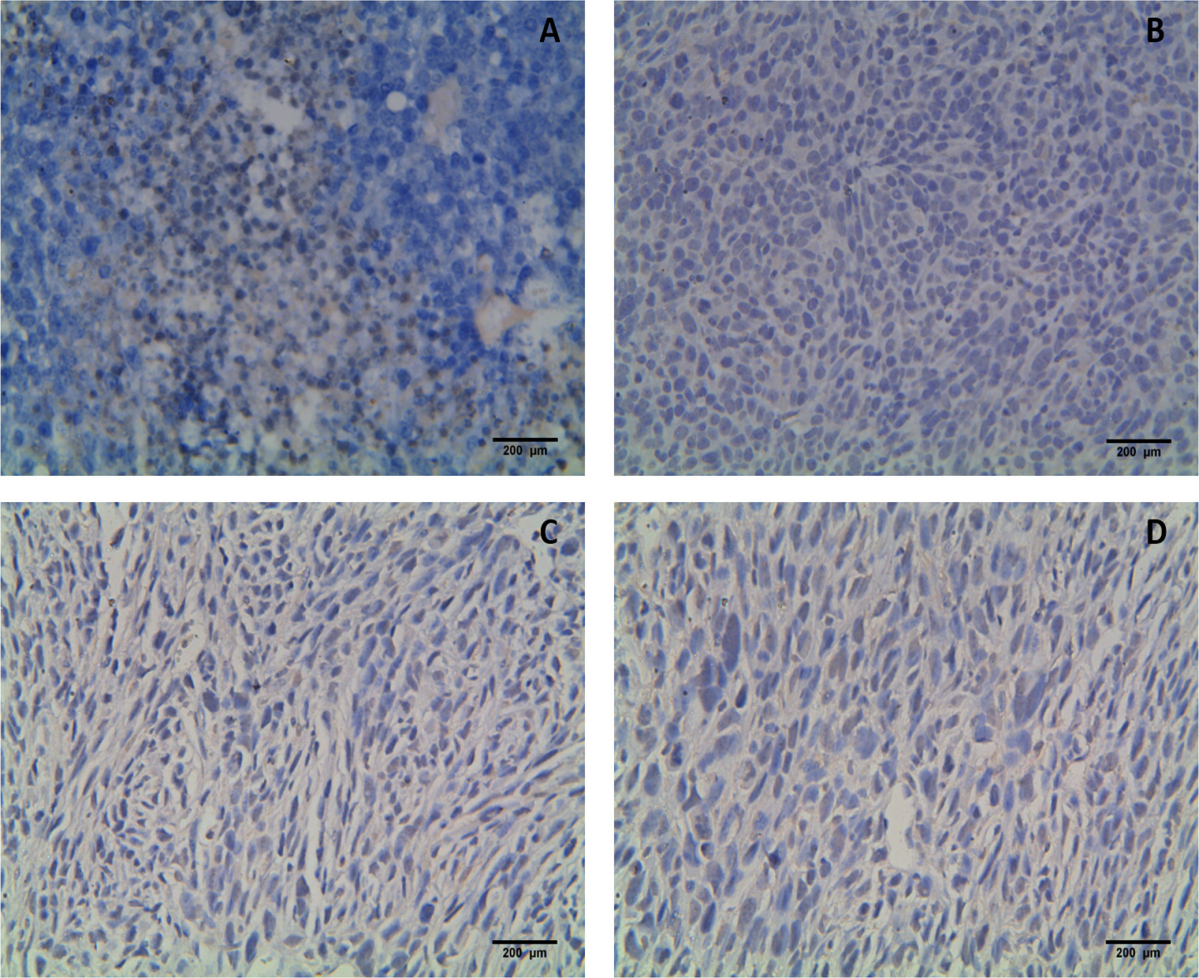
HPV16L1 protein cannot be found in the result of immunohistochemistry Fig 6-A shows results for the positive control: IHC results of TC-1-HPV16 L1, which express HPV16 L1. Fig 6-B shows results for the negative control: TC-1 cells. Fig 6-C and D show IHC results of C3 and C3-luc. Although faint positive signals were observed, the expression of PV16 L1 was negative.

After C3 or C3-luc cells formed tumors in mice, the expression of HPV16 L1 was assayed by immunohistochemistry (IHC). IHC results are shown in Fig 6. TC-1-HPV16 L1 cells, which express HPV16 L1 proteins, were used as a positive control (Fig 6A), and TC-1 cells, which do not express the L1 gene, was used as a negative control (Fig 6B). Although faint positive signals were observed in immunohistochemical stains of C3 and C3-luc cells (Fig 6C and 6D), the expression of PV16 L1 was negative.

### Short tandem repeat analysis

**Fig 7:**
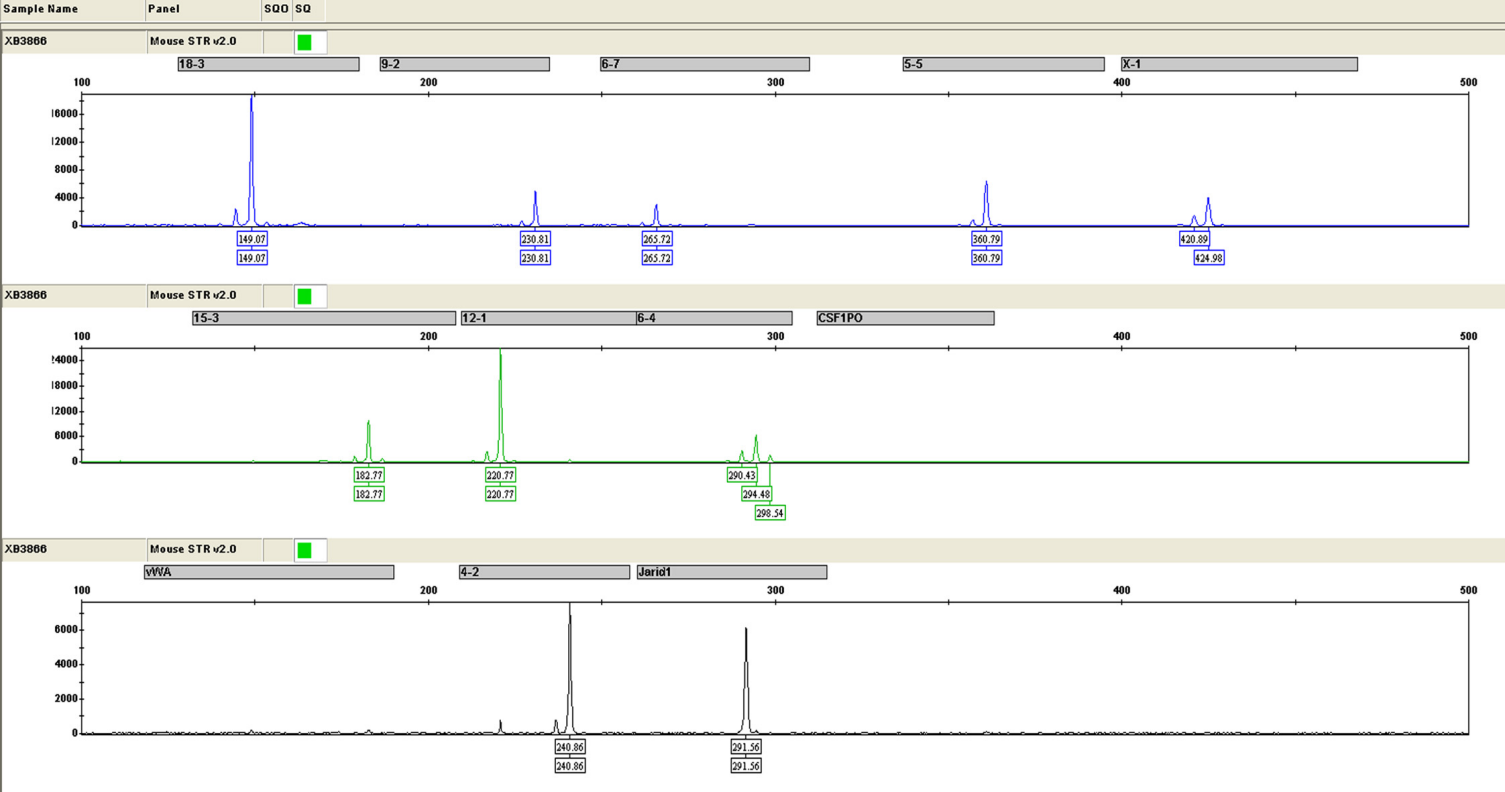
Results of STR analysis. STR results indicate that cell samples were derived from mice and not contaminated by other sources.

All positive and negative control samples provided expected results in the following experiments. The fragment size of STR loci of C3 is shown in Fig 7. Gene Mapper v3.2 software (ABI) was used to analyze the sequences of STR loci. Maps of C3 were well resolved and genotyping results were positive after DNA amplification. These results indicate that cell samples were derived from mice and not contaminated by other sources.

### Screening and identification of C3-luc

**Fig 8:**
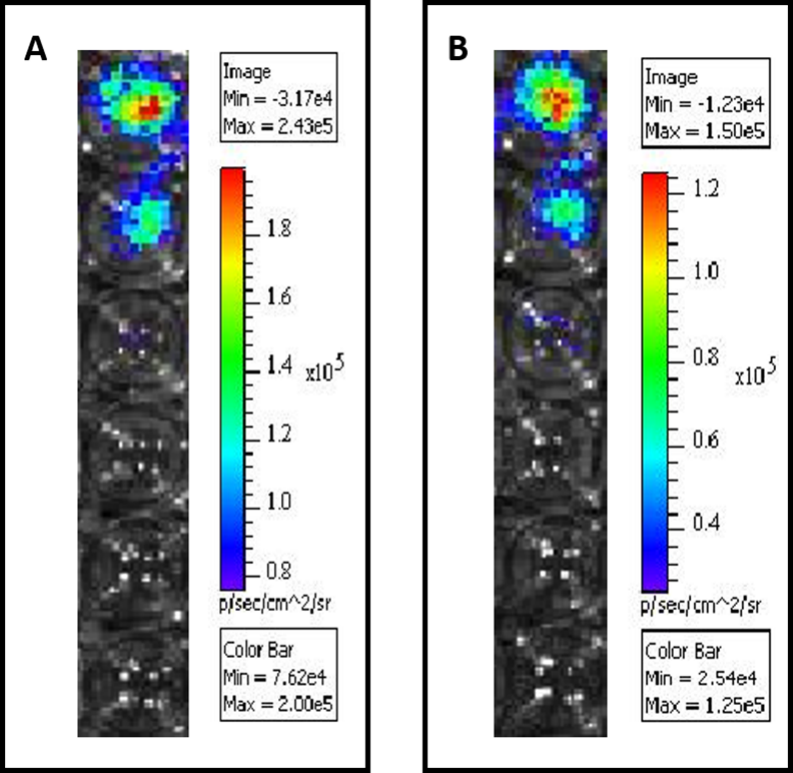
C3-luc cultured in medium without blasticidin. (A) Shows cells after 5 passages and (B) shows cells after 10 passages. Emission of 5.658 × 10^5^ and 5.799 × 10^5^ photons was observed per 10^4^ cells. The results showed that the exogenous gene luciferase can be stably expressed in C3-luc without blasticidin within at least 10 generations.

The optimum concentration of blasticidin is 500 μg/mL, as determined in a preliminary experiment. At day 8, 16, 24, or 30 of blasticidin pressure screening, C3-luc cells were imaged on the IVIS. Per 10^4^ cells, the expression of luciferase increased as time of pressure screening increased, and photon levels measured at day 8, 16, 24, and 30 were 3.365 × 10^4^, 5.823 × 10^4^, 1.766 × 10^5^, and 1.972 × 10^5^, respectively. After 24 days of screening, photon emission was no longer increasing, and cells were screened for 30 days as the next step of the experiment.C3-luc cells were transferred into DMEM (10% FCS and 1% penicillin and streptomycin) without blasticidin after being screened for 30 days. Fig 8A and 8B show cells at passages 5 and 10; these cells expressed luciferase, causing D-luciferin to release photons that were captured by the imaging system. The results showed that the exogenous luciferase gene can be stably expressed in C3-luc without blasticidin within 10 generations.

### Effect of exogenous luciferase on cell growth

**Fig 9:**
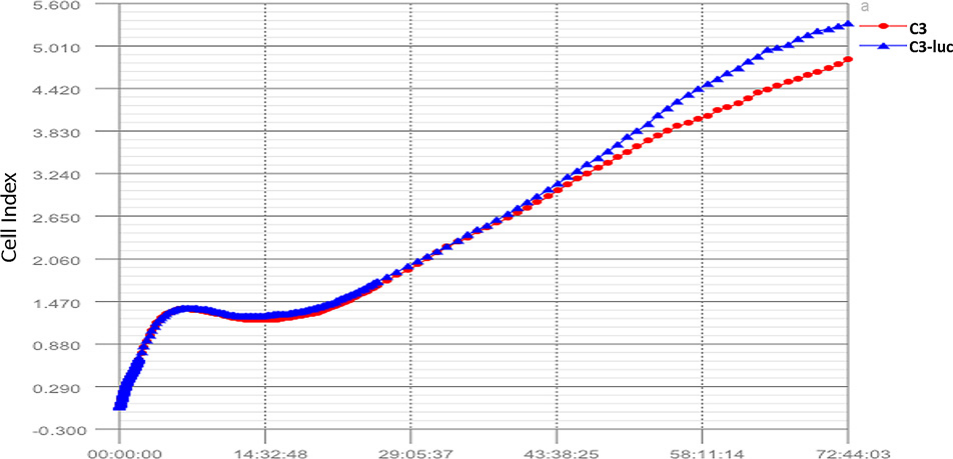
Growth curves for C3 and C3-luc. C3 or C3-luc cells (5 × 10^3^) were added to the wells of an E-PlateL8. The plate was inserted into the iCELLigence to monitor the adherent rate (cell index, CI) of the cells. Both types of cells increased 50-fold over 72 h. Growth rates, adhesion, and proliferation characteristics showed no significant differences. Luciferase addition had no effect on cell growth.

Fig 9 shows the CI, which indicates cell adherence. The CI value is proportional to the number of adherent cells, reflecting proliferation.

As shown in Fig 9, the CI rose from 0.1 to 5.0 in 72 h, indicating that the number of cells increased 50-fold in 72 h. Based on this calculation, the number of cells increased from 5 × 10^3^ to 2.5 × 10^5^. The curves representing the two cell types (C3 and C3-luc) coincided substantially and showed no significant differences in growth rate, adhesion, or proliferation characteristics.

### C3 and C3-luc can be killed as target cells *ex vivo*

For *in vitro* killing experiments, C3 and mouse spleen lymphocytes alone were added to two separate wells of the E-Plate L8. C3 or C3-luc and various sources of mouse spleen lymphocytes were added to the other four wells of the E-Plate L8. C3 or C3-luc, as the targeted model tumor cells expressing HPV16L1, were expected to be killed by mouse lymphocytes that had been immunized with an HPV16L1 vaccine *in vitro*. In this study, the iCELLigence system was used to monitor attachment of cells to identify the sensitivity of C3 or C3-luc cells to lymphocytes, as dead cells detach from wells. In Fig 1, Line 1 shows the process of proliferation of C3 cells; cell number peaked and then declined through the 168 h, which is consistent with the behavior of adherent cells during normal growth in a fixed culture system. Because spleen lymphocytes grown *in vitro* do not show substantial adherence or proliferation, the curve of Line 6 is a straight line. When spleen lymphocytes of the vaccine group (line 4 and 5) and C3 or C3-luc encounter each other under the stimulation of peptides, the peak of tumor cell proliferation arrives earlier and its amplitude is lower due to the anti-proliferative effect. At the same time, there was almost no difference between C3 and C3-luc. The slopes of curves for spleen lymphocytes in PBS (line 2) or DNA/MVA without HPV16 L1 (line 3) and C3-luc as a control differ from the vaccine group and C3 cells alone. The slope of the curve for the PBS group was lower than that for single C3 cells because SPLs were added to compete for medium nutrients. The rationale for the lower slope of the curve for the DNA/MVA without HPV16 L1 group than the PBS group but a higher slope than for the vaccine group may be due to the activation of specific killer cells by DNA or MVA, which may result in non-specific killing of tumor cells. Notwithstanding, the role of SPLs of the vaccine groups in inhibiting tumor cells were more significant.

### Tumors

Formation of palpable tumors required approximately 6–30 d after the cells were transferred into the mice. Fig 2 shows that the greater the number of cells inoculated, the faster the tumors were generated. However, time to tumor formation showed no significant differences between mice inoculated with the same number of C3 or C3-luc cells. Thus, the luciferase gene did not affect the amount of cells and time needed to form tumors.

**Fig 10:**
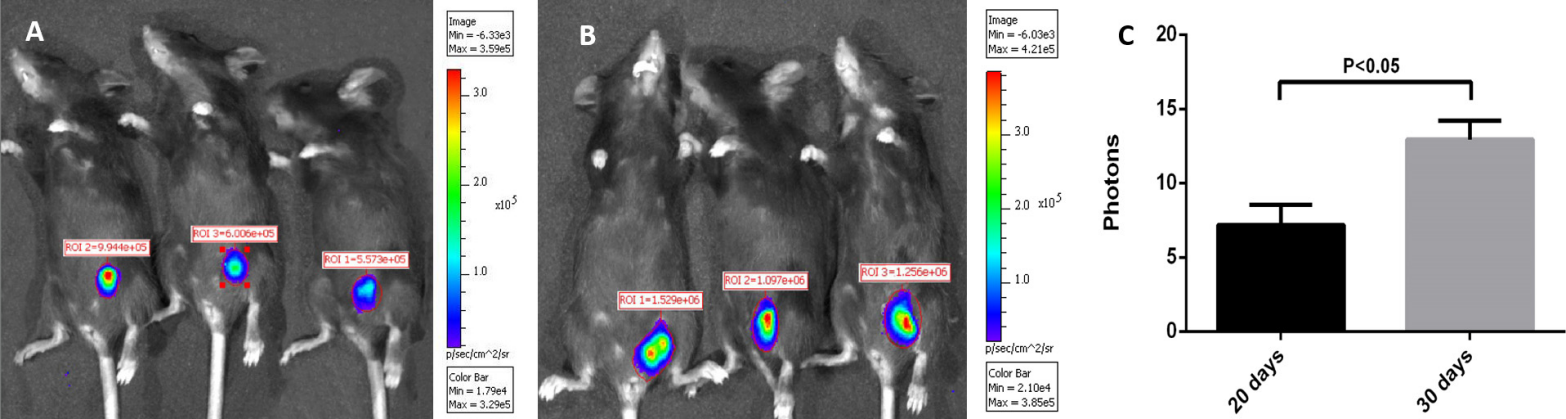
IVIS images of 3 mice 20 d and 30 d after subcutaneous injection of 2 × 10^7^ C3-luc cells. Chloral hydrate (10%) was intraperitoneally injected at 100 μL/mouse to anesthetize the mice. D-luciferin at a concentration of 150 mg/kg in sterile phosphate-buffered saline was injected after they were fully anesthetized. Animals were imaged for 180 s at a binning value of 8 and an FVO of 12.8 cm, 10 min after the injection of D-luciferin. Statistical analysis of photon levels was performed using an unpaired t-test (P < 0.05). Results of statistical analysis indicated that tumor size increased with time, and the correlation was significant.

After 2 × 10^7^ C3-luc cells were transplanted into mice, palpable tumors were detectable after approximately one week. Using the IVIS system, tumors were observed at 20 and 30 d (Fig 10). After mice were anesthetized, D-luciferin was intraperitoneally injected. D-luciferin and luciferase expressed by the C3-luc cells migrated to the tumor site via the blood circulation, interacted with each other, and released fluorescence. The IVIS system can capture the fluorescence and form an image. The number of photons captured by the system can be calculated by software for finding tumors and evaluating tumor size. As shown in Fig 10, tumors were located in the groin, consistent with the position of the injection. At day 20, the number of photons (representing the size of the tumor) detected was 9.94 × 10^5^, 5.57 × 10^5^, 6.01 × 10^5^. At day 30, 15.29 × 10^5^, 10.97 × 10^5^, 12.56 × 10^5^ photons were detected in the same three mice. The difference between tumor sizes at the two time points was significant (P < 0.05).

### C3-Luc tumors are suppressed in mice treated with a vaccine against HPV16L1

**Fig 11:**
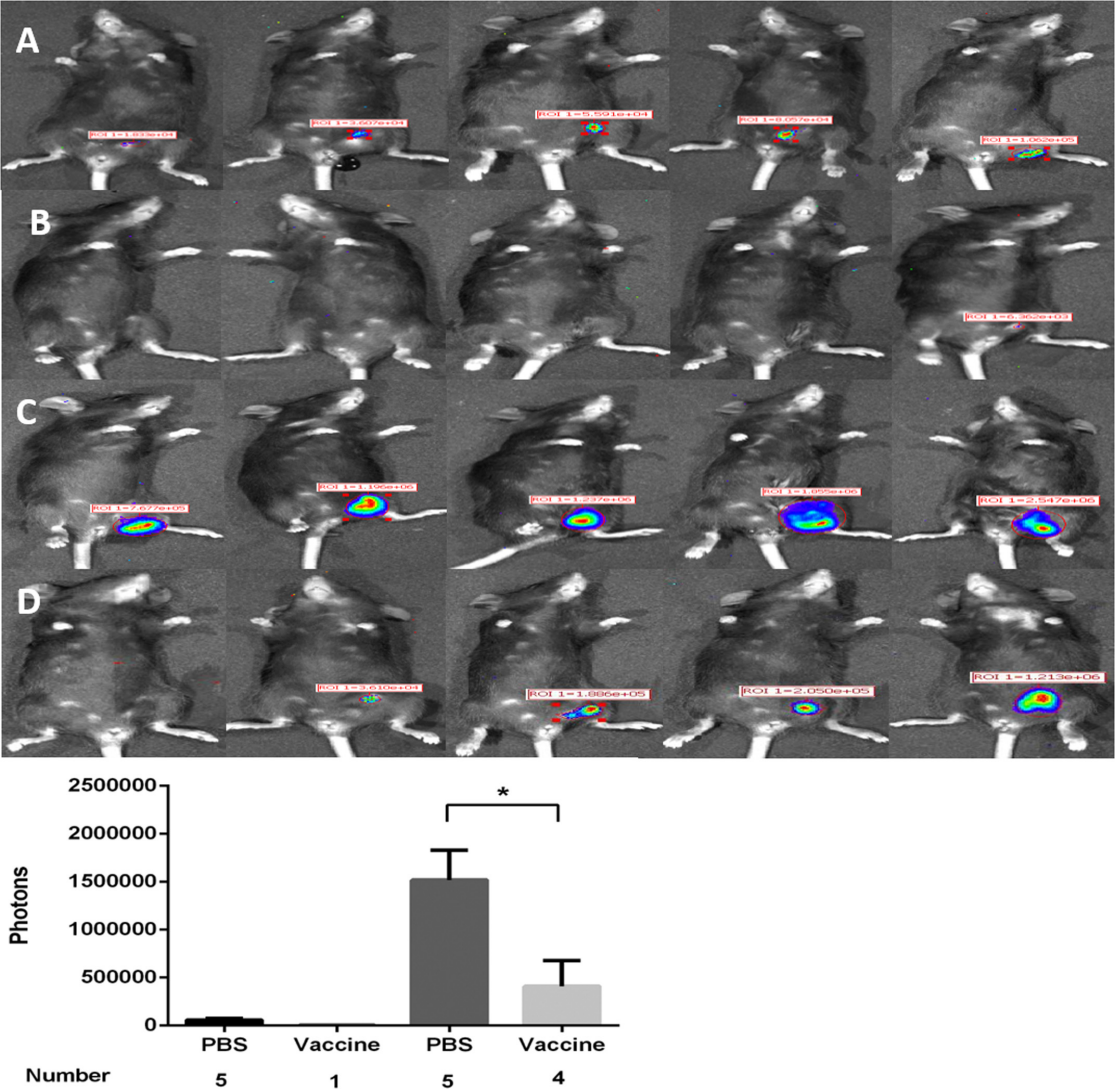
Protective effect of vaccine (primed with DNA-HPV16L1 and boosted with MVA-HPV16L1) in tumor challenge. Ten 6–8 week-old female C57BL/6 mice were divided into 2 groups. One group was primed with pVR16-HPV16L1 (DNA vaccine) and boosted with MVA-HPV16L1 (virus vaccine) and the other group was injected with the same volumes of PBS. C3-luc cells (2 × 10^7^) were subcutaneously injected one week later. (A) and (B) show mice observed using the IVIS system at 10 days after tumor challenge and (C) and (D) show mice observed at 30 d. In the PBS group, 100% of mice developed a tumor in the groin; however, in the vaccine group, only 20% had a visible tumor at 10 d. On day 30, tumor incidence was 100% in the PBS group and had risen to 80% in the vaccine group. The difference in the average number of photons, which represents tumor size, was significant at 30 d. Thus, in *in vivo* experiments, C3-luc cells were sensitive to the HPV16L1 vaccine.

10 C57BL/6 mice were primed with a DNA vaccine and boosted with MVA-HPV16L1 or PBS (5:5). C3-luc cells (2 × 10^7^) were transplanted into mice 1 week after vaccination and mice were imaged using the IVIS system at 10 and 30 days after cells injection. All mice in the PBS group (100%) were positive for tumors (Fig 11A) whereas in the vaccinated group only 1 of 5 (20%) showed tumors (Fig 11B) in the first 10 days. Over the next 20 d, 100% of mice in the PBS group and 80% of mice in the vaccine group showed tumors (Fig 11C and 11D). However, tumor size differed significantly between the PBS and vaccine groups (P < 0.05, Fig 11E). Thirty days after cell transplantation, tumors were larger in the PBS group (P < 0.01).

## Discussion

The genome of C3 cells contains the HPV16 early (E) and late (L) genes. In the life cycle of the HPV virus, E proteins (E1, E2, E4, E5, E6, and E7) play a role in virus replication and cell transformation, and the late (L1 and L2) proteins form the structural units of the viral capsid [17]. The expression of L proteins in virus-infected cells is regulated by the cell cycle and the state of cell differentiation. Although C3 cells are used as models to determine the immune effect of vaccines based on HPV16L1, little is known about the expression of L1 in C3 cells or its mechanism. Previous reports showed that although the HPV16 L1 gene could be detected in C3 cells, HPV16L1 protein detection is nontrivial using conventional methods. This state of L1 is consistent with the tumors coursed by HPV infection. [18]. Therefore, C3 cells are a good model system for investigating potential treatments for HPV infection or for potential tumor vaccines.

In this study, PCR and RT-PCR were employed to determine whether the HPV16L1 genes are present in C3 cells and whether they are transcribed. Western blot and immunohistochemical analyses were also conducted to quantify HPV16L1 protein. Both HPV16L1 DNA and mRNA were detected, indicating that C3 cells carry and transcribe the HPV16 L1 gene. However, L1 protein could not be detected using conventional methods. Previous studies have reported similar results [19, 20]. This posed several questions: 1) Is the expression of L1 suppressed in C3? 2) Is the expression of L1 so low that it is below the detection range of western blotting and immunohistochemistry? 3) Is L1 expression very unstable in C3? 4) Is the L1 antigen presented at the cell surface by MHC class I? We were able to answer some of these questions. First, in *in vitro* experiments, the growth of C3 cells was significantly inhibited by spleen lymphocytes of immunized mice, and the extent of inhibition was closely related to the number of cells. Under real time imaging microscopy, lysis of C3 cells by spleen lymphocytes was recorded. In tumor challenge tests, tumors were found in 20% of the vaccine group, but tumors were found in 100% of the PBS group 10 days after injection of C3. At 30 days, the tumor incidence rate of the vaccine group had increased to 80%, but tumor volume was significantly lower than in the PBS group (P < 0.05). These results all support the hypothesis that HPV16L1 can be expressed and presented at the cell surface and effectively activated cytotoxic T lymphocytes, eventually leading to lysis of C3 cells in the *in vitro* cytotoxicity test and inhibiting tumor growth in the *in vivo* and *in vitro* experiments. The innate immune response and natural killer cells cannot kill tumor cells so efficiently, and thus these results cannot be attributed to these immune responses.

In addition, in order to observe the location and volume of the tumors caused by the C3 cells and calculate their growth rate, the firefly luciferase gene was integrated into the genome of C3 cells. Bioluminescence imaging is a highly efficient technique to detect and quantify enzyme-labeled target sites in living animals [21]. Currently, several companies have developed animal *in vivo* imaging systems to facilitate observation of model cells in experimental animals. When the substrate, luciferin, reaches tumor cells labeled with luciferase via the blood circulation, luciferin is oxidized to oxyluciferin. During the oxidation process, biological fluorescence is emitted (bioluminescence). The IVIS imaging system can detect and record these light signals and analyze the number of photons discharged for the purpose of quantification. However, we needed to exclude the possibility that the expression of the luciferase gene would affect the growth, proliferation, or tumorigenesis of C3 cells, or that it would influence the expression or presentation of the L1 protein. We performed a series of comparative analyses of C3-luc and C3 cells to determine cell growth and proliferation rate, the number of cells required for tumor formation, the time required for the formation of tumors, and whether they could be killed by immunized mouse spleen lymphocytes. All of the results indicated that the addition and expression of the luciferase gene had no significant effect on the characteristics of C3 cells.

Compared with our previous experiments using TC-1 cells, C3 cells showed slow growth and required more cells and more time to form tumors. Slow growth avoids one disadvantage of malignant tumor cells, which proliferate too quickly to determine the efficacy of drugs or vaccines.

In conclusion, the results of our study provide evidence that the HPV16L1 gene is present and that its protein is expressed in C3 cells. Thus, C3 (C3-luc) cells provide a suitable model for research on vaccines that target HPV16L1.
